# 554. Discharge Antibiotic Prescribing at Children’s Hospitals with Established Antimicrobial Stewardship Programs

**DOI:** 10.1093/ofid/ofac492.607

**Published:** 2022-12-15

**Authors:** Rebecca Same, Giyoung Lee, Jared Olson, Brendan Bettinger, Adam Hersh, Matthew Kronman, Jason Newland, Jeffrey S Gerber

**Affiliations:** Children's Hospital of Philadelphia, Philadelphia, Pennsylvania; Center for Pediatric Clinical Effectiveness, Children’s Hospital of Philadelphia, Philadelphia, Pennsylvania; Primary Children's Hospital, Salt Lake City, Utah; Seattle Children's, Seattle, Washington; University of Utah, Salt Lake City, Utah; Seattle Children's Hospital / University of Washington, Seattle, Washington; Washington University School of Medicine, Saint Louis, Missouri; Children's Hospital of Philadelphia, Philadelphia, Pennsylvania

## Abstract

**Background:**

Antibiotic stewardship programs optimize antibiotic use in hospitalized children, but most do not routinely review antibiotic prescriptions at discharge. Up to 30% of discharged children receive additional days of antibiotics, and one single-center study found that 27% of discharge prescriptions were suboptimal.

**Methods:**

We conducted a retrospective cohort study to evaluate duration of therapy (DOT) and antibiotic choice for children < 18 years admitted to 4 children’s hospitals from January 1, 2019 - December 31, 2019 and prescribed antibiotics at discharge for uncomplicated community-acquired pneumonia (CAP), skin and soft tissue infection (SSTI), or urinary tract infection (UTI). We excluded children with complex medical conditions, > 1 infection requiring antibiotics, > 7 day hospital stay, or intensive care unit stay. The primary outcomes were the percentage of subjects prescribed optimal (1) total (inpatient plus outpatient) DOT (4-6 days for CAP and SSTI, ≤8 days for UTI), and (2) antibiotic choice (CAP: amoxicillin; SSTI: clindamycin, amoxicillin-clavulanate, cephalexin, or trimethoprim-sulfamethoxazole (TMP/SMX); UTI: cephalexin, amoxicillin, amoxicillin-clavulanate, TMP/SMX, or nitrofurantoin) based on current national guidelines and available evidence.

**Results:**

2105 encounters were included: 783 CAP, 916 SSTI, and 406 UTI. Median age was 4 years and 49% were female. DOT for each condition are shown in Figure 1 and antibiotic choice in Figure 2. Antibiotic choice was optimal for 66% with CAP, 98% with SSTI, and 88% with UTI. DOT was optimal for 11% with CAP, 4% with SSTI, and 21% with UTI. Both DOT and choice were optimal for 2% with CAP, 4% with SSTI, and 19% with UTI. For all indications, antibiotic choice was optimal for 84% and DOT was optimal for 10%, while only 6% of antibiotic courses included both optimal DOT and antibiotic choice.

Total duration of therapy for 2105 children discharged with antibiotics for community-acquired pneumonia, urinary tract infection, and skin and soft tissue infection.

**Figure 1.**
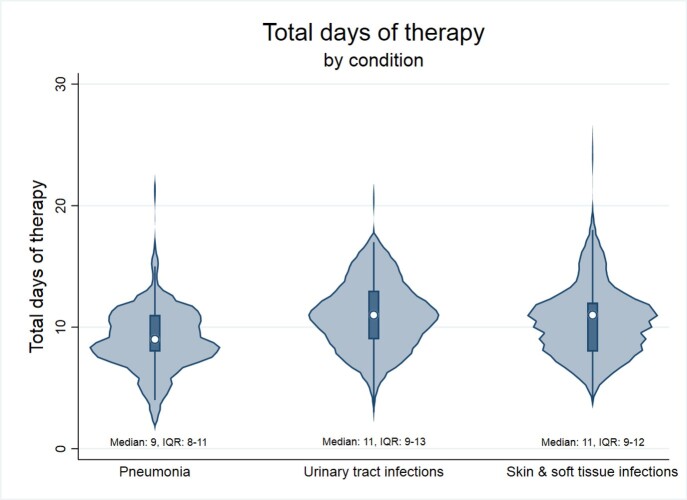
Median duration of antibiotic therapy prescribed for 783 children with community-acquired pneumonia, 406 with urinary tract infection, and 916 with skin and soft tissue infection. The box and whiskers within each plot depict the median (white dot), interquartile range (box) and 5th/95th percentiles (whiskers); the width of each plot represents the number of courses that received that value for overall antibiotic duration.

Discharge antibiotic choices for 2105 children with community-acquired pneumonia, urinary tract infection, and skin and soft tissue infection.

**Figure 2.**
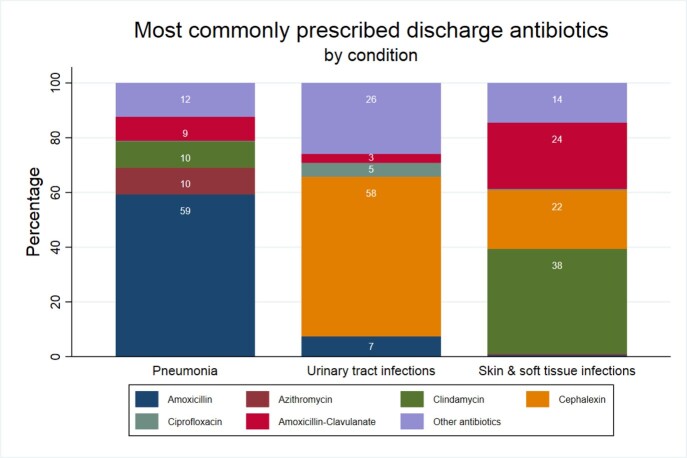
The most commonly prescribed antibiotics at discharge for 2105 children with community-acquired pneumonia, urinary tract infection, or skin and soft tissue infection. Percentages based on total number of antibiotic prescriptions. Other antibiotics include: Cefadroxil, Cefdinir, Cefixime, Doxycycline, Levofloxacin, Linezolid, Nitrofurantoin, trimethoprim-sulfamethoxazole.

**Conclusion:**

At 4 children’s hospitals with established antimicrobial stewardship programs, 94% of discharge antibiotic courses for CAP, UTI, and SSTI were suboptimal either by choice of antibiotic or duration of therapy. Discharge antibiotic prescribing represents a significant opportunity to improve antibiotic use in children.

**Disclosures:**

**Jason Newland, MD**, AHRQ: Grant/Research Support|Merck: Grant/Research Support|NIH: Grant/Research Support|PEW Charitable Trust: Grant/Research Support|Pfizer: Grant/Research Support.

